# Supplementary material for: A distinct Acyl-CoA binding protein (ACBP6) shapes tissue plasticity during nutrient adaptation in Drosophila
Source: Nat Commun. 2023 Nov 21;14:7599. doi: 10.1038/s41467-023-43362-4 (PMC10663470; doi:10.1038/s41467-023-43362-4)
Supplement: Supplementary file 1 — Supplementary Information [file 41467_2023_43362_MOESM1_ESM.pdf]

Supplemental information

A distinct Acyl-CoA binding protein (ACBP6) shapes tissue plasticity during nutrient adaptation in *Drosophila*

Xiaotong Li and Jason Karpac

Supplementary Table 1

Supplementary Figure 1-11

Supplementary Table 1: qRT-PCR Primers

Primer	Sequence (5’-3’)
Acbp6-F	ATGCCCACCTTTGAGGAGA
Acbp6-R	ACTGGGGAGCGTACTTCTTG
Actin5C F	CTCGCCACTTGCGTTTACAGT
Actin5C R	TCCATATCGTCCCAGTTGGTC
Cpt1-F	GCCTATCATGGTTCAGGGCA
Cpt1-R	TGCCCTGAACCATGATAGGC
Gpat4-F	ATGTTACGTCGCGCTTCGAG
Gpat4-R	CGTTGGCCAATACCAACGA
Lipin-F	GTCCGGTACGAAGAAGTCCG
Lipin-R	TCTGAGATACGGCAACTGCT
Mtpβ-F	CAGGCACTCGCTTTTGTCAT
Mtpβ-R	CCTGGCAATGTTGGAGGTCT
Nd51-F	CAGCGGTATGAAGTGGTCCT
Nd51-R	TGTAGAACTCGCCACGAATG
SdhA-F	CATGTACGACACGGTCAAGG
SdhA-R	GACCAGTACGATCAGCCACA
Upd1-F	AATCAGCTGAAGCGCCACG
Upd1-R	GGAATTGGGCTTGAGCTTGG
Upd2-F	CCACAACCTGCGACTCTTCT
Upd2-R	GCGCGGTGGGTTATATCTT
Upd3-F	GAGCACCAAGACTCTGGACA
Upd3-R	CCAGTGCAACTTGATGTTGC
Acbp1-F	GGGGGATTGCAATACAGATAAGC
Acbp1-R	GCGTTTGACGACAACTGAGAC
Acbp2-F	TGTGGCAAACAACACACAACA
Acbp2-R	TTGCCCTTCTGCTTGTTCCA
Acbp3-F	GCCTTTTCACCGCATTACCA
Acbp3-R	GGAGAGACCCTTGTTGGAGC
Acbp4-F	GCCGAACTCGCCAAGAACT
Acbp4-R	TTGGAGAGACCCTTGTGGGC
Acbp5-F	AATATGGCCGATTTCAACGC
Acbp5-R	TCAGCCAGGCATCGTACTTAG
Fasn1-F	AGCTAATAACGGCAGTCAACGG
Fasn1-R	CAGGTTTAGTTGTAGGGGCTAGA

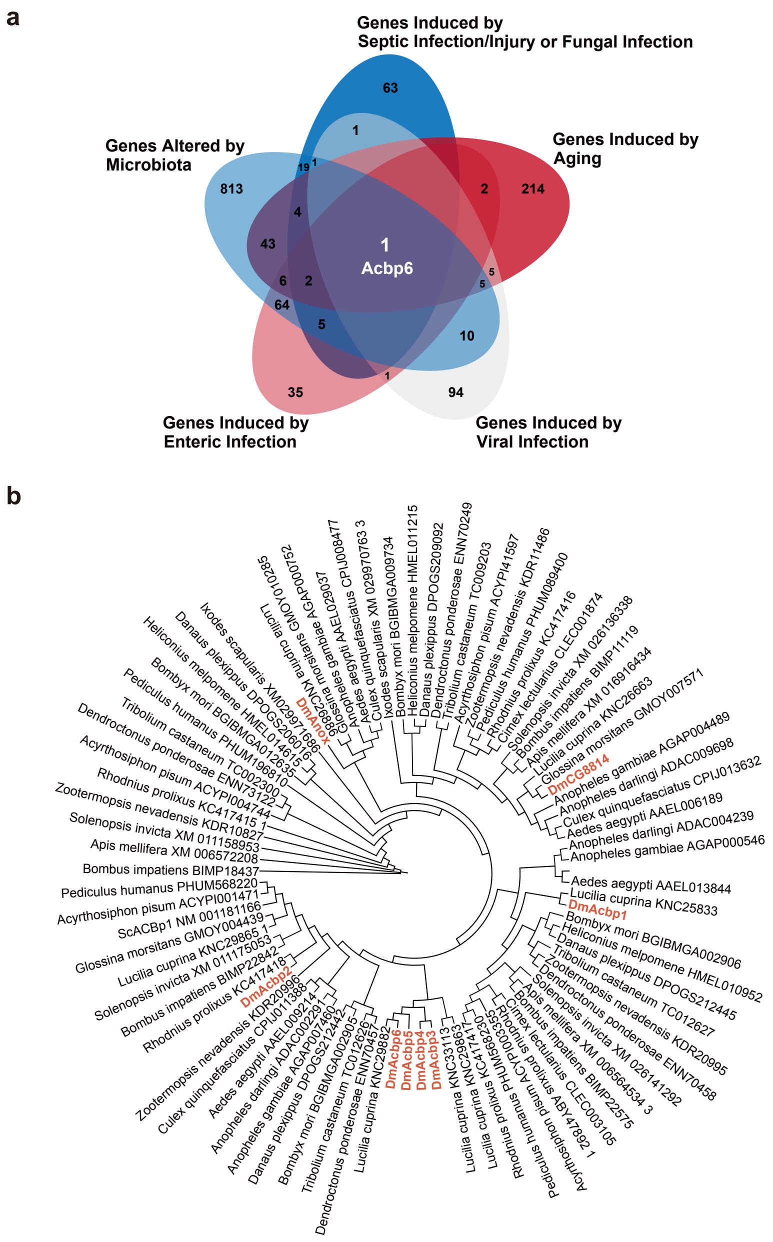

Supplementary Figure 1. Acbp6 was generated by gene expansion in the *Drosophila* genus with specialized expression in the midgut

(a) Venn Diagram highlighting overlap of upregulated genes from meta-analysis of various *Drosophila melanogaster* transcriptomic databases (labeled in various colors). Significantly upregulated genes (with a cut-off at ≥ 2-fold up-regulated) were selected from each dataset to use in the analysis, and up-regulated genes common to more than one group were analyzed and compared by using ImageGP. The single overlapping gene in all datasets is Acbp6. (b) Phylogenetic tree of insect Acbp family of genes. *Acbp* genes within the *Drosophila melanogaster* genome are marked in red.

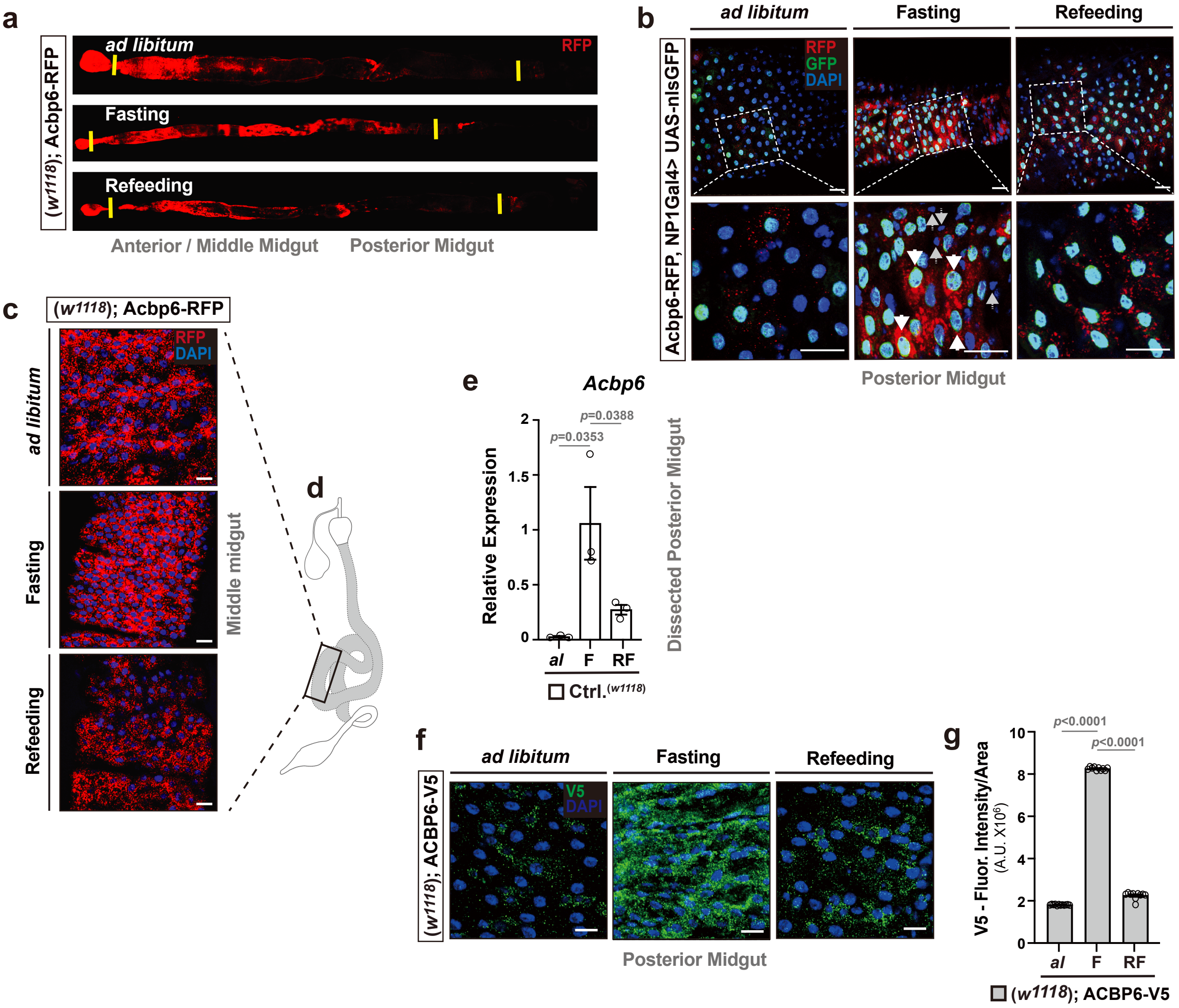

Supplementary Figure 2. Regulation of Acbp6 in the *Drosophila* midgut during nutrient adaptation

(a) Comprehensive visualization of Acbp6 expression patterns, indicating by Acbp6-RFP reporter, in the fly gut during nutrient adaptation (anterior and posterior guidelines marked in yellow). (b) Acbp6 expression pattern in dissected posterior midguts from *w1118*; NP1Gal4, UAS-nls-GFP/Acbp6-RFP transgenic flies during nutrient adaptation; stained with DAPI (blue); RFP (red) and GFP (green). White arrows highlight overlap of RFP signal (Acbp6-RFP) with GFP signal (enterocyte-specific GFP induction) during fasting. Grey arrows represent small (non-enterocyte) GFP- and RFP-negative cells. (c) Acbp6 expression pattern in dissected middle midguts from *w1118*; Acbp6-RFP/Acbp6-RFP transgenic flies during nutrient adaptation; stained with DAPI (blue); RFP (red). n = 3 independent experiments in (a-c). (d) Model of gastrointestinal tract depicting location of Acbp6 reporter activity. (e)

Transcriptional changes (measured by qRT-PCR) of *Acbp6* in dissected posterior midguts during nutrient adaptation. n=3 independent samples. Genotypes; *w1118*. (f) Nutrient-dependent changes of ACBP6 protein levels ($ACBP6^{V5}$), indicated by V5 immunostaining of dissected posterior midguts in $Acbp6^{P}$-$ACBP6^{V5}$ transgenic flies, stained with anti-V5 (green) and DAPI (nuclei, blue). Genotypes; *w1118*; $Acbp6^{P}$-$ACBP6^{V5}$/ $Acbp6^{P}$-$ACBP6^{V5}$. $Acbp6^{P}$ represent an endogenous promoter/enhancer region. (g) Quantification of V5 immunostains (fluor. intensity per field, posterior midgut; A.U. [Arbitrary Units]; from left to right, bars represent n=12, 10 and 10 independent samples) during nutrient adaptation. Genotypes; *w1118*; $Acbp6^{P}$-$ACBP6^{V5}$/ $Acbp6^{P}$-$ACBP6^{V5}$.

Bars represent mean ± SEM (unpaired 2-tailed Student's *t*-test). The exact *p* values are provided in figure. Source data are provided as a Source Data file. *ad libitum*, *al*; Fasting (F) represents 2 days of nutrient deprivation, and Refeeding (RF) represents 2 days of refeeding unless otherwise stated. Scale bars, 10 μm.

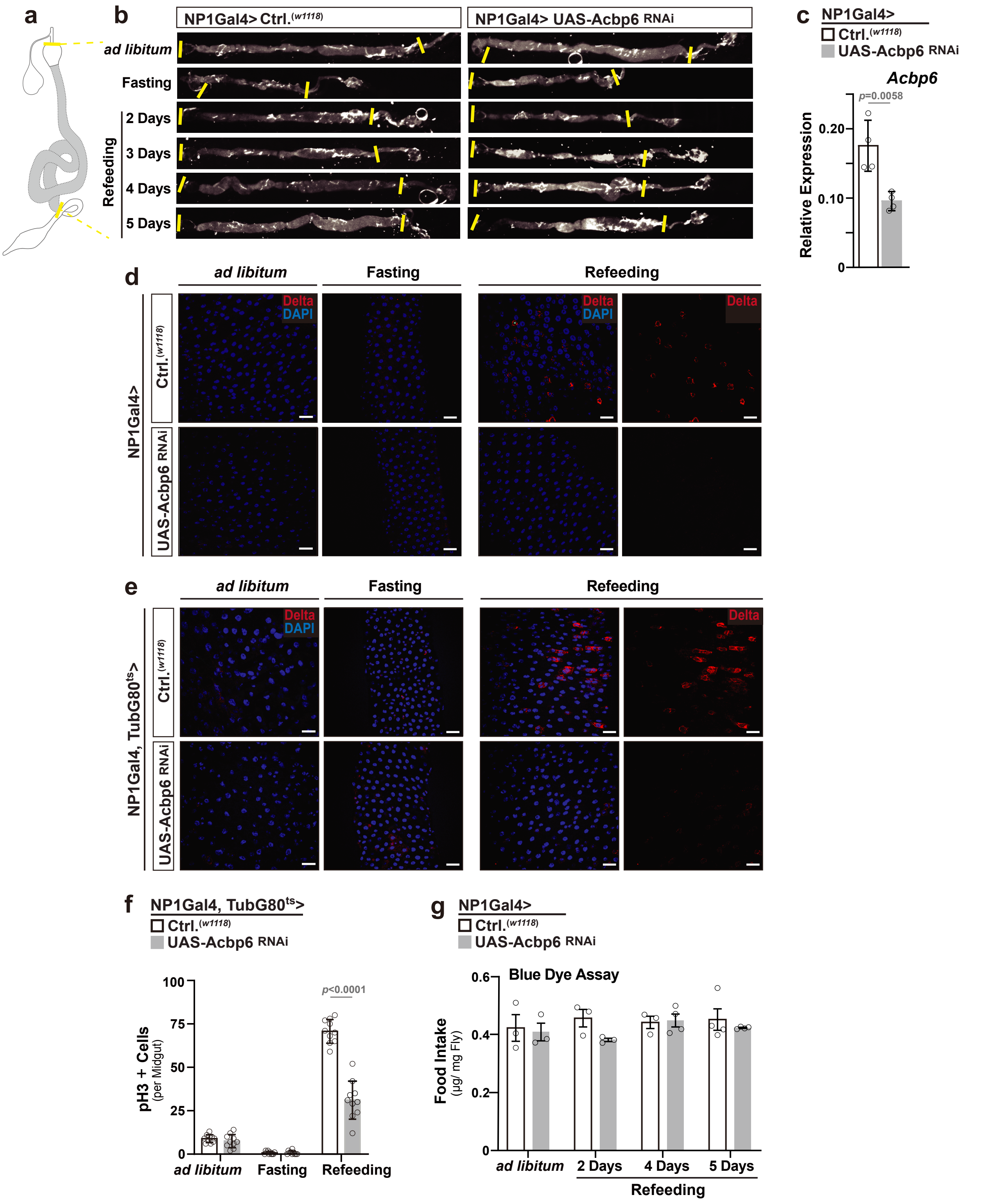

Supplementary Figure 3. Distinct spatio-temporal control of Acbp6 function dictates midgut tissue plasticity during nutrient adaptation by regulating proliferative homeostasis

(a) Model of gastrointestinal tract depicting gut length measurement. (b) Representative

images of midgut length changes (anterior and posterior guidelines marked in yellow and in the model in panel [a]). (c) Transcriptional changes (measured by qRT-PCR) of *Acbp6* in dissected whole midguts upon enterocyte-specific depletion of Acbp6 (fed *ad libitum*). n = 4 independent samples. Genotypes; *w1118*; NP1Gal4/+ (controls, Ctrl.) and *w1118*; NP1Gal4/UAS-Acbp6 RNAi. (d) Representative images of Delta positive cells in dissected posterior midguts (anti-Delta [red] and DAPI [nuclei; blue]) upon enterocyte-specific depletion of Acbp6 during nutrient adaptation. Genotypes; *w1118*; NP1Gal4/+ (controls, Ctrl.) and *w1118*; NP1Gal4/UAS-Acbp6 RNAi. (e) Immunostaining to detect Delta positive cells in dissected posterior midguts upon adult-specific and enterocyte-specific depletion of Acbp6 during nutrient adaptation; anti-Delta (red) and DAPI (nuclei; blue). Genotypes; *w1118*; NP1Gal4, TubGal80^{ts}/+ (controls, Ctrl.) and *w1118*; NP1Gal4, TubGal80^{ts}/UAS-Acbp6 RNAi. (f) Quantification of phospho-Histone (H3) positive cells (per dissected whole midgut, n=10 independent samples) upon adult-specific and enterocyte-specific depletion of Acbp6 during nutrient adaptation. Genotypes; *w1118*; NP1Gal4, TubGal80^{ts}/+ (controls, Ctrl.) and *w1118*; NP1Gal4, TubGal80^{ts}/UAS-Acbp6 RNAi. (g) Feeding behavior (assayed by a Blue Dye Assay) of flies upon enterocyte-specific depletion of Acbp6 during *ad libitum* feeding, or during refeeding (2 days, 4 days, or 5 days) after fasting (2-days) (from left to right, bars represent n=3, 3, 3, 3, 3, 3, 4 and 4 independent samples). Genotypes; *w1118*; NP1Gal4/+ (controls, Ctrl.) and *w1118*; NP1Gal4/UAS-Acbp6 RNAi.

Bars represent mean ± SEM (unpaired 2-tailed Student's *t*-test). The exact *p* values are provided in figure. Source data are provided as a Source Data file. Fasting represents 2 days of nutrient deprivation, and Refeeding represents 2 days of refeeding unless otherwise stated. Scale bars, 10 μm.

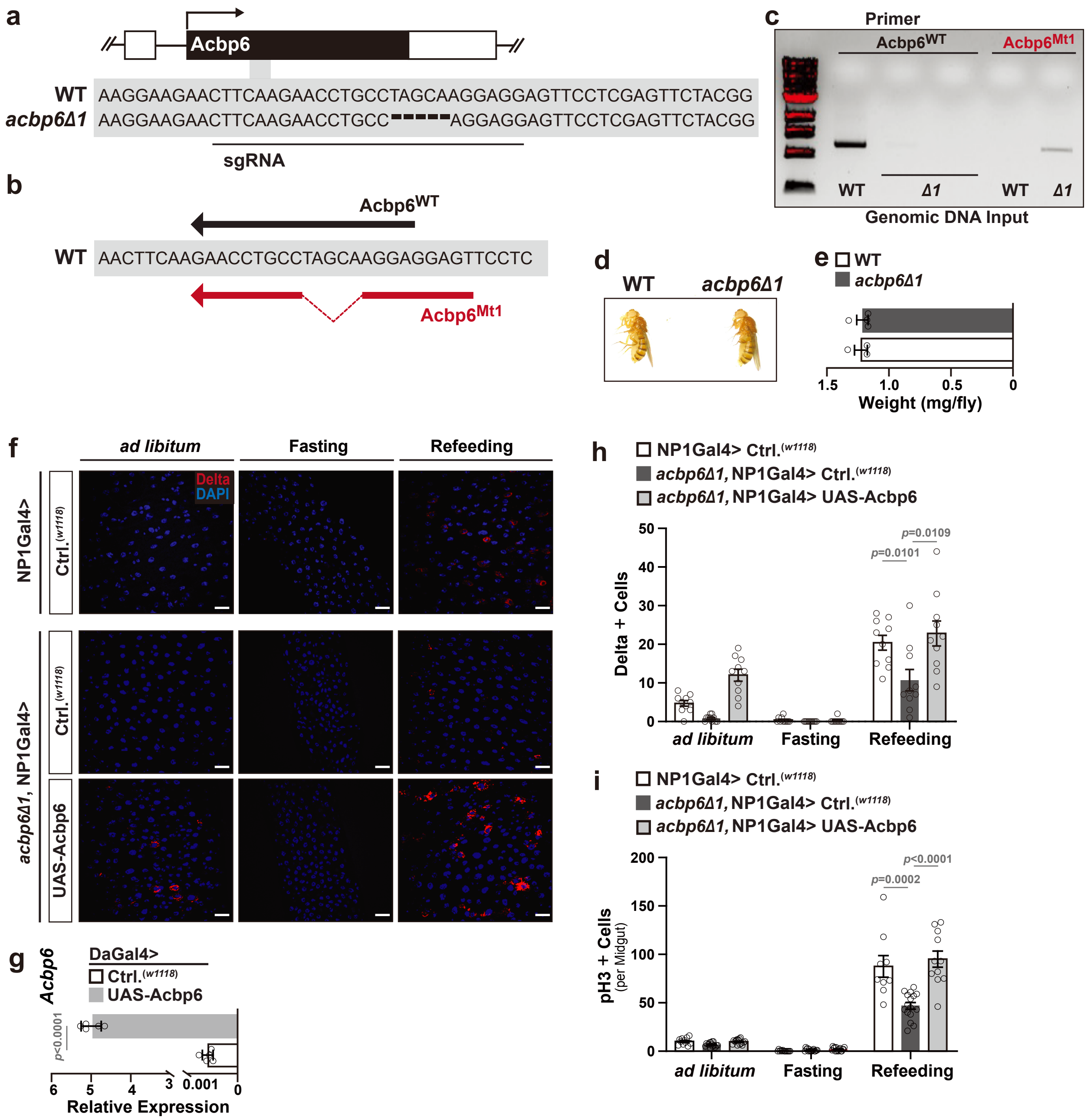

Supplementary Figure 4. Generation of *acbp6* mutant flies for loss-of-function analysis

(a-c) Generation and validation of *acbp6* mutant flies using CRISPR-Cas9. (a) Acbp6 gene locus, highlighting wild-type (WT) coding sequence, *acbp6Δ1* deletion, and guide RNA overlap sequence (sgRNA). (b) Schematic of primers used for mutant validation. (c) Gel image displaying PCR products from wild-type or mutant (*acbp6Δ1*) genomic template utilizing Acbp6WT or Mt1 primers. (d) Representative image of Acbp6 mutant flies. (e) Wet weight measurement of wild-type and mutant (*acbp6Δ1*) flies. n=3 (WT) and 4 (*acbp6Δ1*) independent replicates (10 flies per replicate). (f) Immunostaining to detect Delta positive

cells in dissected posterior midguts in *acbp6* mutant flies (*acbp6Δ1*), or with enterocyte-specific Acbp6 rescue in the mutant background, during nutrient adaptation; anti-Delta (red) and DAPI (nuclei; blue). Genotypes; *w1118*; NP1Gal4/+ (controls, Ctrl.) and *w1118*; NP1Gal4/+; *acbp6Δ1*/+ and *w1118*; NP1Gal4/UAS-Acbp6; *acbp6Δ1*/+. (g) Transcriptional changes (measured by qRT-PCR) of *Acbp6* in whole flies (fed *ad libitum*). n=5 replicates. Genotypes; *w1118*;; Daughterless (Da)Gal4/+ (controls, Ctrl.) and *w1118*; UAS-Acbp6/+; DaGal4/+. (h-i) Quantification of (h) Delta positive cells (per field, posterior midgut; n=10 independent samples) and (i) phospho-Histone (H3) positive cells (per dissected whole midgut, from left to right, bars represent n= 11, 15, 15, 13, 15, 16, 9, 16 and 11 independent samples), in *acbp6* mutant flies (*acbp6Δ1*), or with enterocyte-specific Acbp6 rescue in the mutant background, during nutrient adaptation. Genotypes; *w1118*; NP1Gal4/+ (controls, Ctrl.) and *w1118*; NP1Gal4/+; *acbp6Δ1*/+ and *w1118*; NP1Gal4/UAS-Acbp6; *acbp6Δ1*/+.

Bars represent mean ± SEM (unpaired 2-tailed Student's *t*-test). The exact *p* values are provided in figure. Source data are provided as a Source Data file. Fasting represents 2 days of nutrient deprivation, and Refeeding represents 2 days of refeeding. Scale bars, 10 μm.

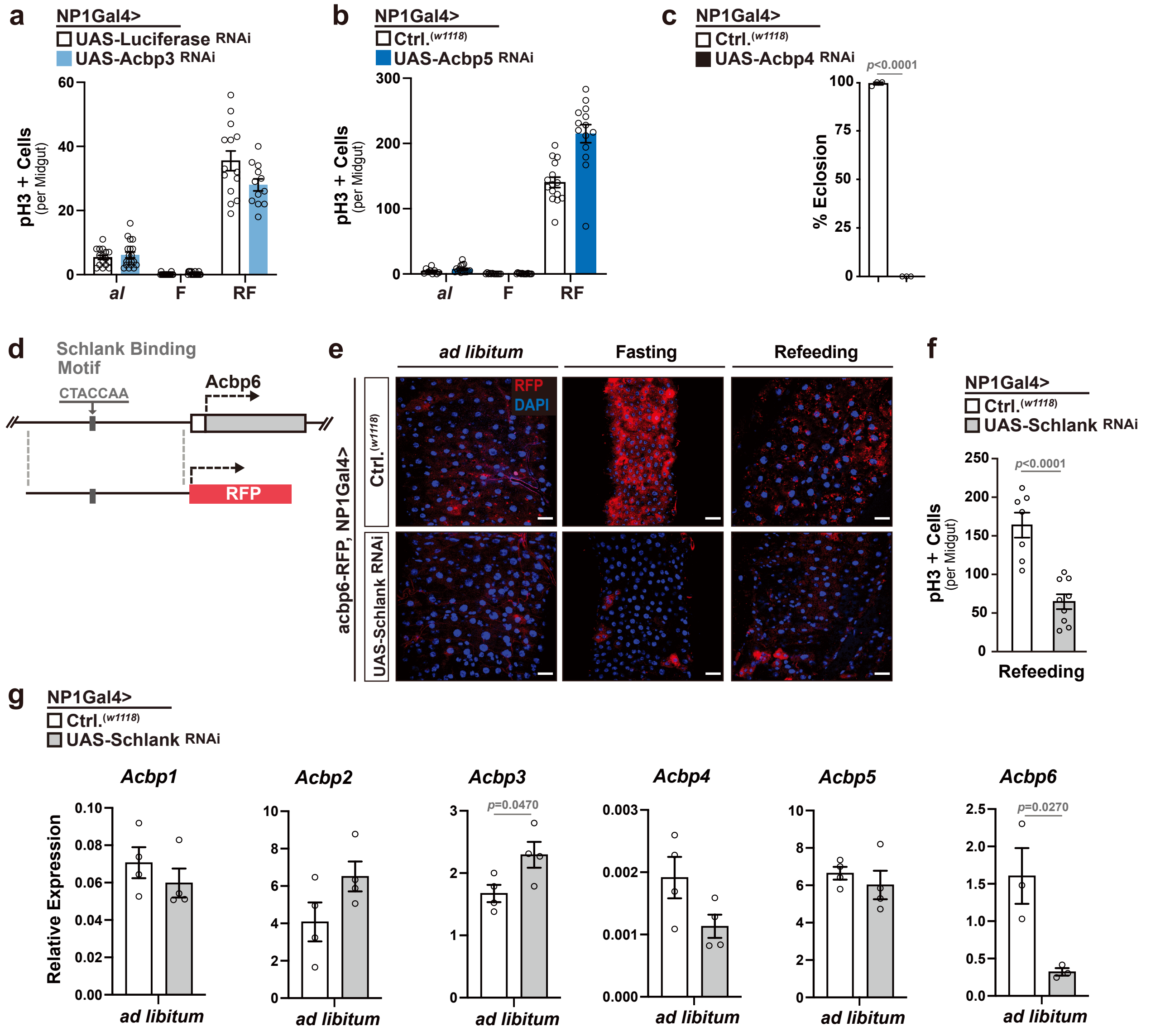

Supplementary Figure 5. Comprehensive analysis of different Acbps' role in ISC proliferation and identification of upstream regulators to Acbp6 during nutrient-dependent tissue remodeling

(a-b) Quantification of phospho-Histone (H3) positive cells (per dissected whole midgut, from left to right, bars represent n= 16, 19, 18, 18, 14, 12; 9, 15, 12, 14, 15 and 14 independent samples) upon enterocyte-specific depletion of Acbp3 or Acbp5 during nutrient adaptation. Genotypes; *w1118*; NP1Gal4/+ (controls, Ctrl.), *w1118/y v*; NP1Gal4/UAS-luciferase RNAi (controls), *w1118/y v*; NP1Gal4/UAS-Acbp3 RNAi, and *w1118*; NP1Gal4/UAS-Acbp5 RNAi. (c) Eclosion rate analysis after Acbp4 knockdown. n= 3 independent experiments. Genotypes; *w1118*; NP1Gal4/+ (controls, Ctrl.), *w1118*; NP1Gal4/UAS-Acbp4 RNAi. (d) Schematic of

Acbp6 promoter region showing Schlank binding motif and Acbp6-RFP reporter construction. (e) Acbp6 expression pattern (acbp6-RFP) in dissected posterior midguts concurrent enterocyte-specific depletion of Schlank during nutrient adaptation; stained with DAPI (blue); RFP (red). Genotypes; *w1118*; acbp6-RFP, NP1Gal4/+ (controls, Ctrl.), *w1118*; acbp6-RFP, NP1Gal4/UAS-Schlank RNAi. (f) Quantification of phospho-Histone (H3) positive cells (per dissected whole midgut, from left to right, bars represent n= 7 and 9 independent samples) upon enterocyte-specific depletion of Schlank during nutrient adaptation. Genotypes; *w1118*; NP1Gal4/+ (controls, Ctrl.) and *w1118*; NP1Gal4/UAS-Schlank RNAi. (g) Transcriptional changes (measured by qRT-PCR) of *Acbp1, Acbp2, Acbp3, Acbp4, Acbp5, Acbp6* in dissected whole midguts (fed *ad libitum*) upon enterocyte-specific depletion of Schlank. n= 4 independent samples for *Acbp1-Acbp5*, and n=3 for *Acbp6*. Genotypes; *w1118*; NP1Gal4/+ (controls, Ctrl.) and *w1118*; NP1Gal4/UAS-Schlank RNAi.

Bars represent mean ± SEM (unpaired 2-tailed Student's *t*-test). The exact *p* values are provided in figure. Source data are provided as a Source Data file. Fasting (F) represents 2 days of nutrient deprivation, and Refeeding (RF) represents 2 days of refeeding. *Ad libitum*; al. Scale bars, 10 μm.

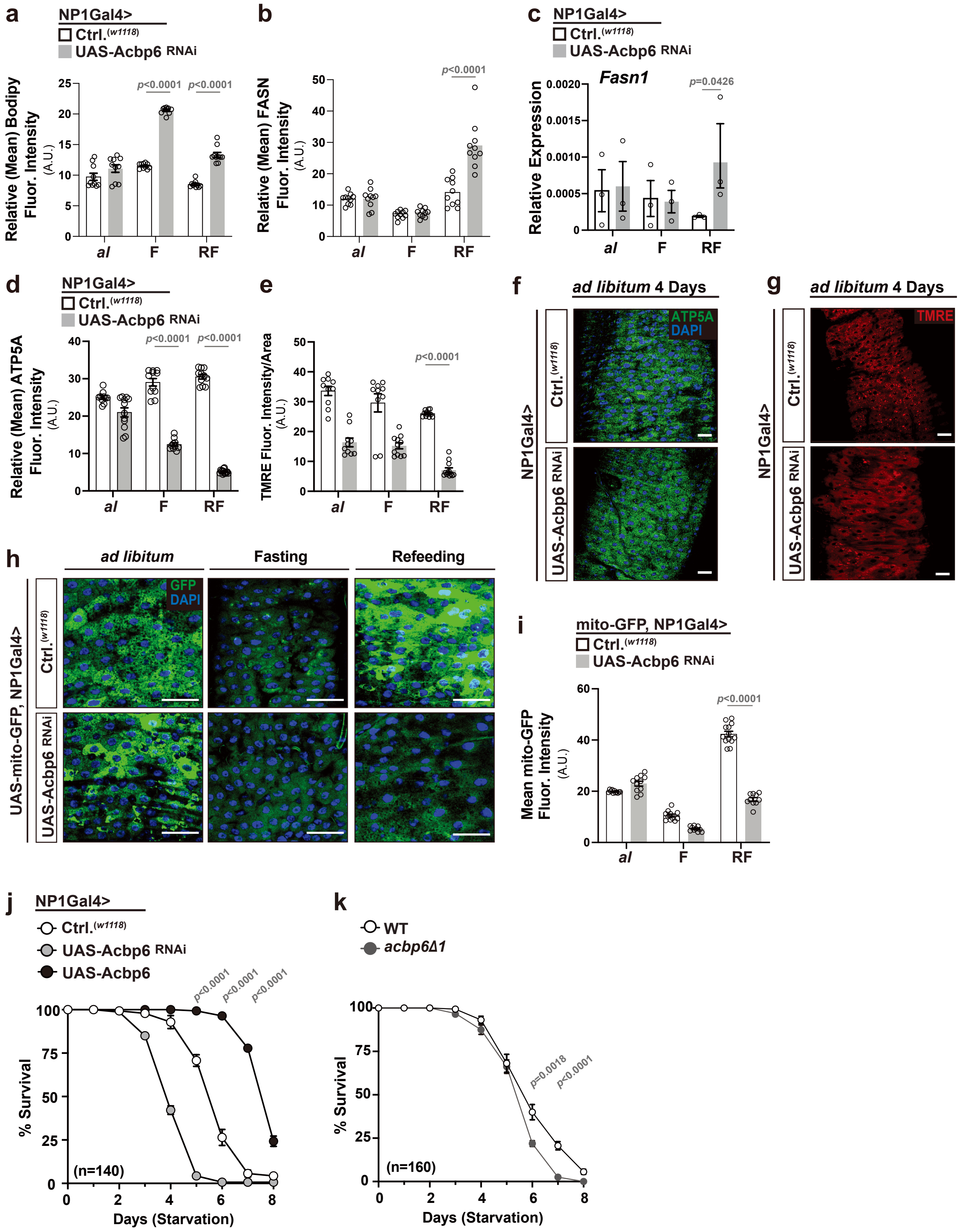

Supplementary Figure 6. Acbp6 loss-of-function induces aberrant switching of nutrient-dependent metabolism in the midgut

(a-b) Quantification of (a) Bodipy and (b) FASN1 immunostains (fluor. intensity per field, posterior midgut; A.U. [Arbitrary Units]; n=10 independent samples) upon enterocyte-specific

depletion of Acbp6 during nutrient adaptation. Genotypes; *w1118*; NP1Gal4/+ (controls, Ctrl.) and *w1118*; NP1Gal4/UAS-Acbp6 RNAi. (c) Transcriptional change (measured by qRT-PCR) of *Fasn1* in dissected whole midguts during nutrient adaptation. n = 3 independent samples. Genotypes; *w1118*; NP1Gal4/+ (controls, Ctrl.) and *w1118*; NP1Gal4/UAS-Acbp6 RNAi. (d-e) Quantification of (d) ATP5a, (e) TMRE immunostains (fluor. intensity per field, posterior midgut; A.U. [Arbitrary Units]; from left to right, bars represent n= 10, 12, 11, 10, 11 and 14 independent samples in (d), and 10, 10, 10, 11, 11 and 12 independent samples in (e)) upon enterocyte-specific depletion of Acbp6 during nutrient adaptation. Genotypes; *w1118*; NP1Gal4/+ (controls, Ctrl.) and *w1118*; NP1Gal4/UAS-Acbp6 RNAi. (f-g) Negative controls showed *ad libitum* feeding for 4 days has no effects on mitochondria dynamics, indicated by ATP5a (f) or TMRE (g) staining. n = 3 independent experiments in (f-g). Genotypes; *w1118*; NP1Gal4/+ (controls, Ctrl.) and *w1118*; NP1Gal4/UAS-Acbp6 RNAi. (h-i) Mitochondria numbers/intensity assessed by mito-GFP upon enterocyte-specific depletion of Acbp6 during nutrient adaptation. Mito-GFP (green) and DAPI (nuclei; blue). In (i), from left to right, bars represent n= 11, 11, 11, 10, 13 and 10 independent samples. Genotypes; *w1118*; NP1Gal4/+; mito-GFP/+ (controls, Ctrl.) and *w1118*; NP1Gal4/UAS-Acbp6 RNAi; mito-GFP/+. (j) Starvation sensitivity assay upon enterocyte-specific depletion or overexpression of Acbp6 (n=140 flies). Genotypes; *w1118*; NP1Gal4/+ (controls, Ctrl.), *w1118*; NP1Gal4/UAS-Acbp6 RNAi and *w1118*; NP1Gal4/UAS-Acbp6. (k) Starvation sensitivity assay in acbp6 mutant flies (*acbp6Δ1*) or controls (wild-type, WT; revertant) (n=160 flies).

Bars represent mean ± SEM (unpaired 2-tailed Student's *t*-test). The exact *p* values are provided in figure. Source data are provided as a Source Data file. Fasting (F) represents 2 days of nutrient deprivation, and Refeeding (RF) represents 2 days of refeeding. *Ad libitum*; al. A.U represents Arbitrary Units. Scale bars, 10 μm.

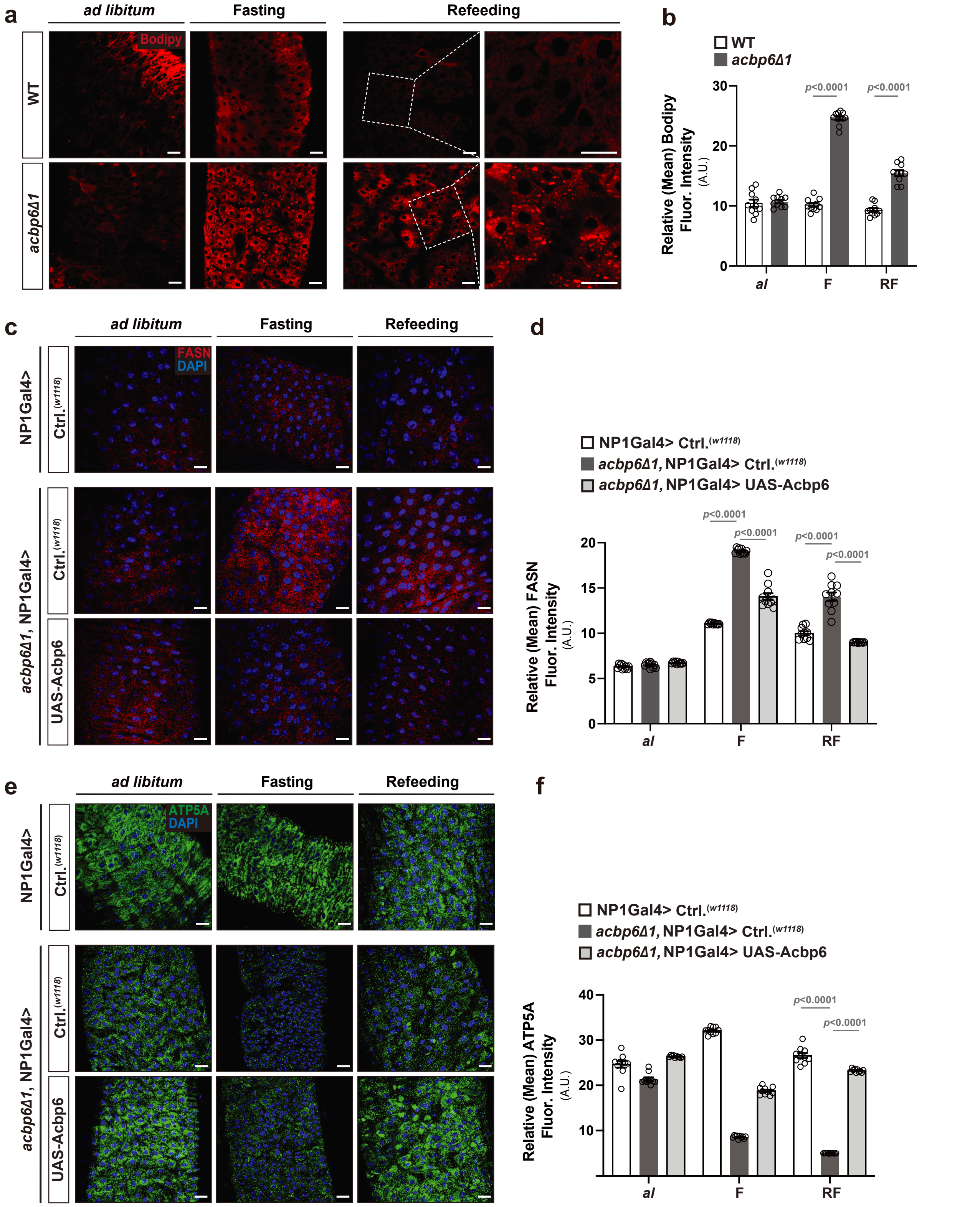

Supplementary Figure 7. Acbp6 loss-of-function induces aberrant lipid syntheses and mitochondria dynamics during nutrient adaptation in the midgut

(a-b) Neutral lipid/lipid droplet fluorescent staining (a) and related quantification (b) (fluor. intensity per field; A.U. [Arbitrary Units]; from left to right, bars represent n= 10, 10, 10, 11,

10 and 10 independent samples) of dissected posterior midguts in *acbp6* mutant flies (*acbp6Δ1*) or controls (wild-type, WT; revertant) during nutrient adaptation; stained with Bodipy (red, reduced state). (c-d) FASN immunostaining (c) and related quantification (d) (fluor. intensity per field; A.U. [Arbitrary Units]; n=10 independent samples) of dissected posterior midguts during nutrient adaptation, stained with anti-FASN (red) and DAPI (nuclei, blue). Genotypes; *w1118*; NP1Gal4/+ (controls, Ctrl.) and *w1118*; NP1Gal4/+; *acbp6Δ1*/+ and *w1118*; NP1Gal4/UAS-Acbp6; *acbp6Δ1*/+. (e-f) Nutrient-dependent changes of mitochondria intensity in *acbp6* mutant flies (*acbp6Δ1*), or with enterocyte-specific Acbp6 rescue in the mutant background, indicating by mitochondrial ATP5a immunostaining of dissected posterior midguts during nutrient adaptation, stained with anti-ATP5A (green) and DAPI (nuclei, blue). In (f), n=10 independent samples. Genotypes; *w1118*; NP1Gal4/+ (controls, Ctrl.) and *w1118*; NP1Gal4/+; *acbp6Δ1*/+ and *w1118*; NP1Gal4/UAS-Acbp6; *acbp6Δ1*/+.

Bars represent mean ± SEM (unpaired 2-tailed Student's *t*-test). The exact *p* values are provided in figure. Source data are provided as a Source Data file. Fasting (F) represents 2 days of nutrient deprivation, and Refeeding (RF) represents 2 days of refeeding. *Ad libitum*; al. A.U represents Arbitrary Units. Scale bars, 10 μm.

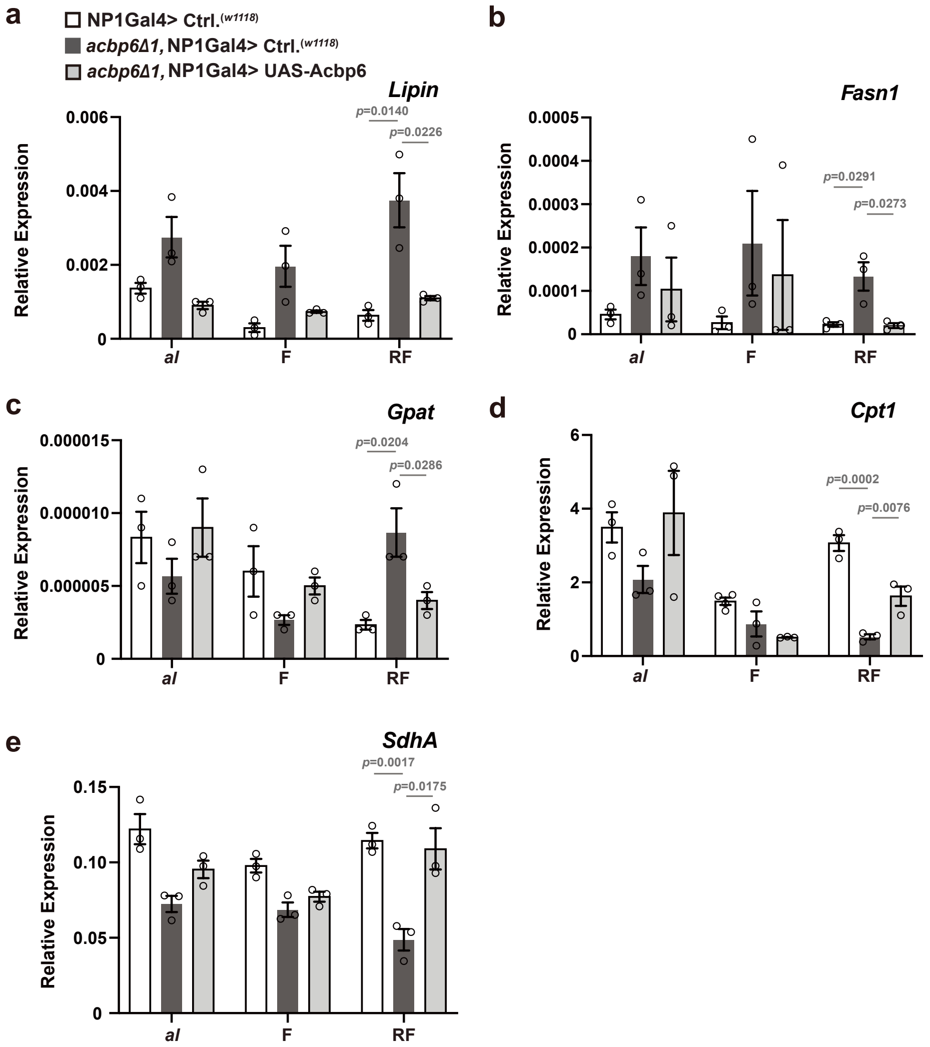

Supplementary Figure 8. Nutrient-dependent transcriptional changes of genes controlling lipid synthesis and mitochondrial homeostasis in the midgut are shaped by Acbp6 function

Nutrient-dependent transcriptional changes ([a] *Lipin*, [b] *Fasn1*, [c] *Gpat*, [d] *Cpt1*, and [e] *SdhA*) in *acbp6* mutant flies (*acbp6Δ1*), or with enterocyte-specific Acbp6 rescue in the mutant background, measured by qRT-PCR (dissected whole midguts, n=3 independent samples) during nutrient adaptation. Genotypes; *w1118*; NP1Gal4/+ (controls, Ctrl.) and *w1118*; NP1Gal4/+; *acbp6Δ1*/+ and *w1118*; NP1Gal4/UAS-Acbp6; *acbp6Δ1*/+*.*

Bars represent mean ± SEM (unpaired 2-tailed Student's *t*-test). The exact *p* values are provided in figure. Source data are provided as a Source Data file. Fasting (F) represents 2 days of nutrient deprivation, and Refeeding (RF) represents 2 days of refeeding. *Ad libitum*; al.

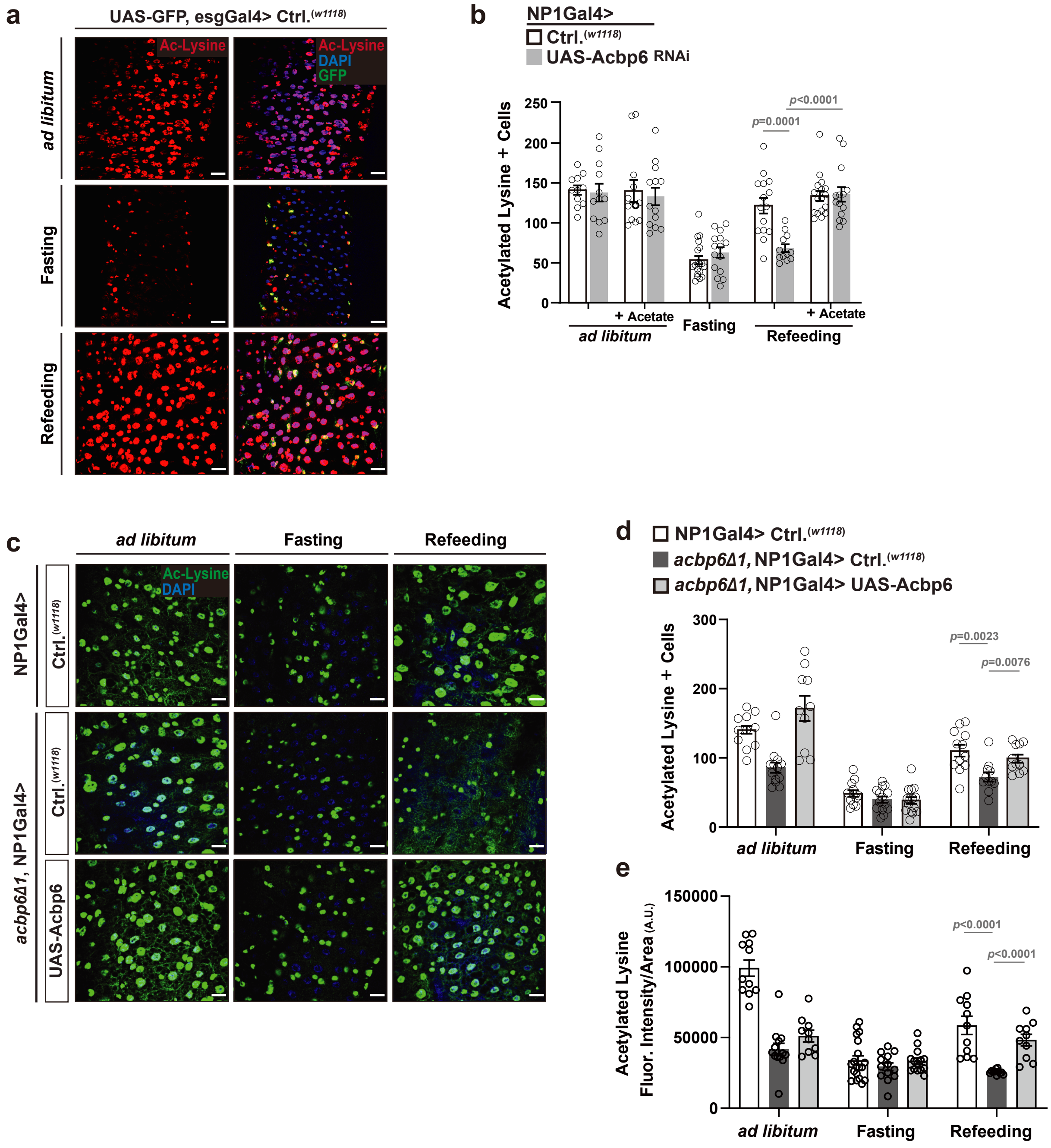

Supplementary Figure 9. Functional analysis of Acbp6 in the control of midgut pan-protein acetylation during nutrient adaptation

(a) Acetylated (Ac)-lysine immunostaining of dissected posterior midguts during nutrient adaptation, highlighting staining across the midgut epithelial layer, including intestinal stem cells and enteroblasts (marked with esgGal4, UAS-GFP); stained with anti-Ac-Lysine (red) and DAPI (nuclei, blue); GFP (green) (n= 3 independent experiments). Genotype; *w1118*; esgGal4, UAS-GFP/+. (b) Quantification of acetylated-lysine positive cells in dissected posterior midguts (per field; from left to right, bars represent n= 11, 12, 12, 13, 19, 15 , 15,

12 , 17, 14 independent samples) during nutrient adaptation (+/-dietary acetate supplementation) upon enterocyte-specific depletion of Acbp6 during nutrient adaptation. Genotypes; w1118; NP1Gal4/+ (controls, Ctrl.) and *w1118*; NP1Gal4/UAS-Acbp6 RNAi. (c) Acetylated (Ac)-lysine immunostaining of dissected posterior in *acbp6* mutant flies (*acbp6Δ1*), or with enterocyte-specific Acbp6 rescue in the mutant background, during nutrient adaptation; stained with anti-Ac-Lysine (green) and DAPI (nuclei, blue). Genotypes; *w1118*; NP1Gal4/+ (controls, Ctrl.) and *w1118*; NP1Gal4/+; *acbp6Δ1*/+ and *w1118*; NP1Gal4/UAS-Acbp6; *acbp6Δ1*/+. (d) Quantification of acetylated-lysine positive cells in dissected posterior midguts (per field; from left to right, bars represent n= 11, 13, 10, 12, 14, 15, 12, 11 and 11 independent samples) during nutrient adaptation from the above genotypes. (e) Quantification of acetylated-lysine fluorescence intensity/area in dissected posterior midguts (per field; A.U. [Arbitrary Units]; from left to right, bars represent n= 11, 13, 10, 19, 13, 14, 11, 11 and 10 independent samples) during nutrient adaptation from the above genotypes.

Bars represent mean ± SEM (unpaired 2-tailed Student's *t*-test). The exact *p* values are provided in figure. Source data are provided as a Source Data file. Fasting represents 2 days of nutrient deprivation, and Refeeding represents 2 days of refeeding. Scale bars, 10 μm.

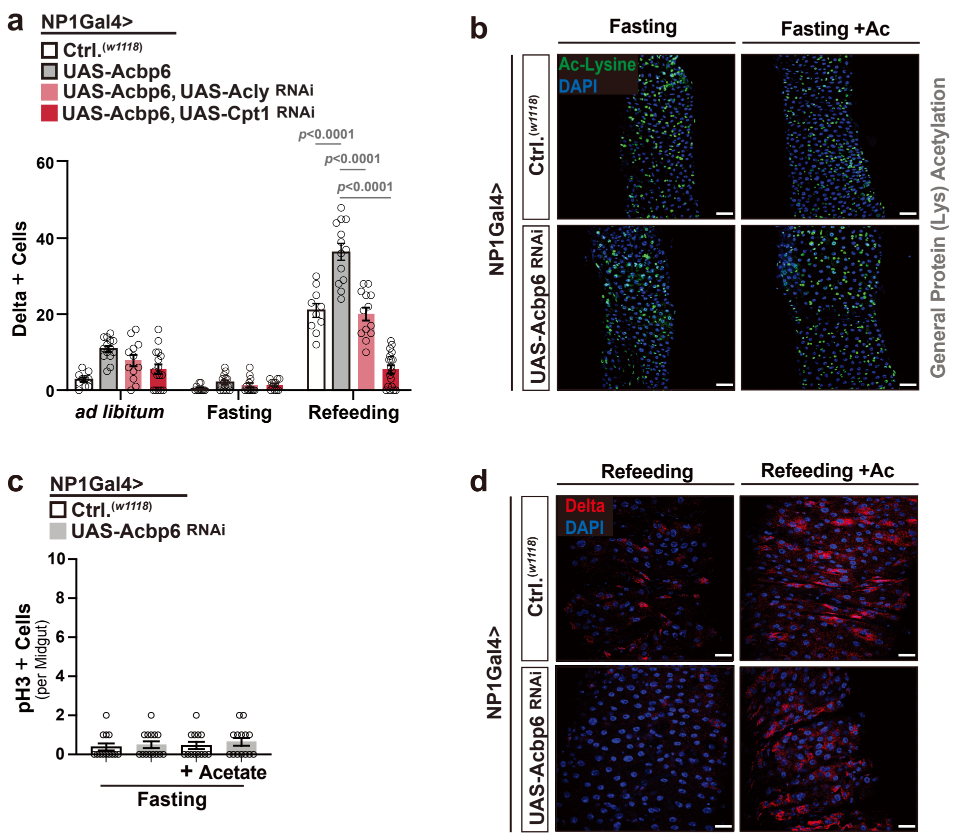

Supplementary Figure 10. Functional analysis of Acbp6 in the control of midgut tissue plasticity during fasting-refeeding transitions

(a) Quantification of Delta positive cells in dissected posterior midguts upon enterocyte-specific upregulation of Acbp6 or upregulation of Acbp6 and concurrent depletion of Acly or Cpt1 during nutrient adaptation, from left to right, bars represent n= 10, 14, 12, 16, 10, 14, 12, 11, 10, 13, 13 and 18 independent samples. Genotypes; *w1118*; NP1Gal4/+ (controls, Ctrl.), and *w1118*; NP1Gal4/UAS-Acbp6, and *w1118*; NP1Gal4, UAS-Acbp6/UAS-Cpt1 RNAi, and *w1118*; NP1Gal4, UAS-Acbp6/UAS-Acly RNAi. (b-c) Effects of dietary acetate supplementation (50 mM) on pan-protein acetylation (posterior midgut) and (b) phospho-Histone (H3) positive cells (c) (per dissected whole midgut, from left to right, bars represent n= 13, 14, 13 and 14 independent samples) in the midgut upon enterocyte-specific depletion of Acbp6 in the posterior midgut during fasting. Genotypes; *w1118*; NP1Gal4/+ (controls, Ctrl.) and *w1118*; NP1Gal4/UAS-Acbp6 RNAi. (d) Effects of dietary acetate supplementation (50 mM) on Delta immunostaining upon enterocyte-specific depletion of Acbp6 in the posterior midgut during refeeding. Genotypes; *w1118*; NP1Gal4/+ (controls, Ctrl.) and *w1118*;

NP1Gal4/UAS-Acbp6 RNAi.

Bars represent mean ± SEM (unpaired 2-tailed Student's *t*-test). The exact *p* values are provided in figure. Source data are provided as a Source Data file. Fasting represents 2 days of nutrient deprivation, and Refeeding represents 2 days of refeeding. Scale bars, 10 μm.

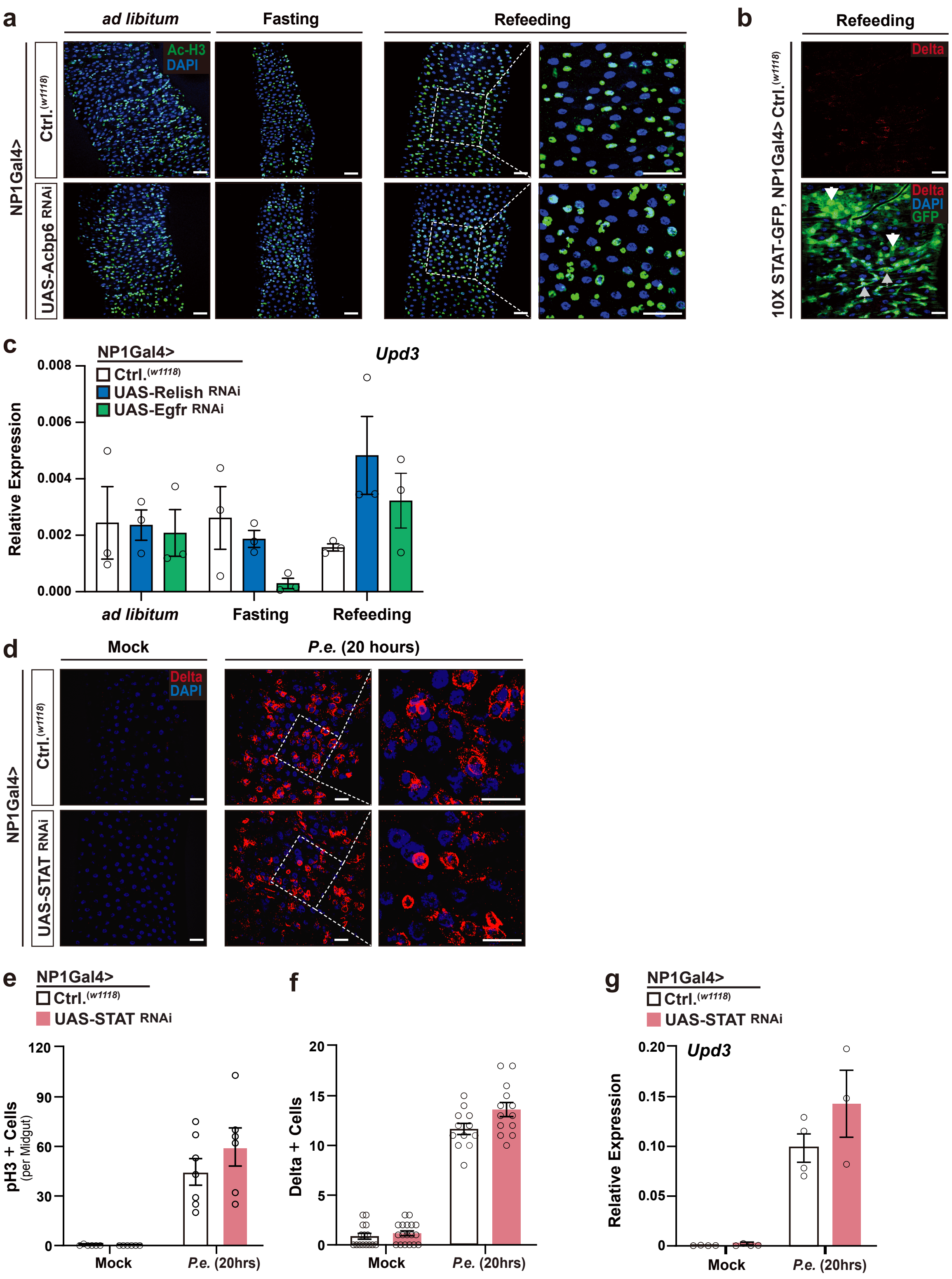

Supplementary Figure 11. Enterocyte-specific Stat92e function is not required for infection-induced midgut regenerative responses

(a) Acetylated (Ac) Histone (H3)-lysine immunostaining of dissected posterior midguts upon enterocyte-specific depletion of Acbp6 during nutrient adaptation, stained with anti-Ac-H3

(green) and DAPI (nuclei, blue) (n= 3 independent experiments). Genotypes; *w1118*; NP1Gal4/+ (controls, Ctrl.) and *w1118*; NP1Gal4/UAS-Acbp6 RNAi. (b) Stat92e activity throughout the epithelial layer (using 10X STAT-GFP transgenic reporter flies) in dissected posterior midguts during refeeding (2 days); stained with DAPI (nuclei, blue) and co-stained with anti-Delta (red); GFP (green) (n= 3 independent experiments). Genotype; *w1118*; 10X STAT-GFP, NP1Gal4/+ (control, Ctrl.). (c) Transcriptional changes (measured by qRT-PCR) of *Upd3* in dissected whole midguts upon enterocyte-specific knockdown of Relish or Egfr during nutrient adaptation. Genotypes; *w1118*; NP1Gal4/+ (controls, Ctrl.), *w1118*; NP1Gal4/UAS-Relish RNAi and *w1118*; NP1Gal4/UAS-Egfr RNAi. n = 3 independent samples. (d) Immunostaining to detect Delta positive cells in dissected posterior midguts upon enterocyte-specific depletion of STAT (Stat92e) after oral infection by *Pseudomonas entomophila* (*P.e*. 20 hrs post-infection; compared to mock treatment). Delta (red) and DAPI (nuclei; blue). Genotypes; *w1118*; NP1Gal4/+ (controls, Ctrl.) and *w1118*; NP1Gal4/UAS-STAT RNAi. (e-f) Quantification of (e) phospho-Histone (H3) positive cells (per dissected whole midgut, from left to right, bars represent n= 6, 6, 7 and 6 independent samples), and (f) Delta positive cells (per field, posterior midgut, from left to right, bars represent n= 15, 18, 12 and 13 independent samples) upon enterocyte-specific depletion of STAT (Stat92e) after oral infection by *Pseudomonas entomophila* (*P.e*. 20hrs post-infection; compared to mock treatment). Genotypes; *w1118*; NP1Gal4/+ (controls, Ctrl.) and *w1118*; NP1Gal4/UAS-STAT RNAi. (g) Transcriptional changes (measured by qRT-PCR) of *Upd3* in dissected whole midguts upon enterocyte-specific depletion of STAT (Stat92e) after oral infection by *Pseudomonas entomophila* (*P.e*. 20 hrs post-infection; compared to mock treatment). Genotypes; *w1118*; NP1Gal4/+ (controls, Ctrl.) and *w1118*; NP1Gal4/UAS-STAT RNAi. From left to right, bars represent n= 4, 4, 4 and 3 independent samples.

Bars represent mean ± SEM (unpaired 2-tailed Student's *t*-test). The exact *p* values are provided in figure. Source data are provided as a Source Data file. Fasting (F) represents 2 days of nutrient deprivation, and Refeeding (RF) represents 2 days of refeeding. *Ad libitum*; al. Scale bars, 10 μm.
