# Supplementary material for: A distinct Acyl-CoA binding protein (ACBP6) shapes tissue plasticity during nutrient adaptation in Drosophila
Source: Nat Commun. 2023 Nov 21;14:7599. doi: 10.1038/s41467-023-43362-4 (PMC10663470; doi:10.1038/s41467-023-43362-4)
Supplement: Supplementary file 3 — Reporting Summary [file 41467_2023_43362_MOESM3_ESM.pdf]

nature portfolio

Corresponding author(s): Jason Karpac

Last updated by author(s): Nov 5, 2023

Reporting Summary

Nature Portfolio wishes to improve the reproducibility of the work that we publish. This form provides structure for consistency and transparency in reporting. For further information on Nature Portfolio policies, see our Editorial Policies and the Editorial Policy Checklist.

Statistics

For all statistical analyses, confirm that the following items are present in the figure legend, table legend, main text, or Methods section.

n/a	Confirmed	
☐	☒	The exact sample size (*n*) for each experimental group/condition, given as a discrete number and unit of measurement
☐	☒	A statement on whether measurements were taken from distinct samples or whether the same sample was measured repeatedly
☐	☒	The statistical test(s) used AND whether they are one- or two-sided<br>*Only common tests should be described solely by name; describe more complex techniques in the Methods section.*
☒	☐	A description of all covariates tested
☐	☒	A description of any assumptions or corrections, such as tests of normality and adjustment for multiple comparisons
☐	☒	A full description of the statistical parameters including central tendency (e.g. means) or other basic estimates (e.g. regression coefficient) AND variation (e.g. standard deviation) or associated estimates of uncertainty (e.g. confidence intervals)
☐	☒	For null hypothesis testing, the test statistic (e.g. *F*, *t*, *r*) with confidence intervals, effect sizes, degrees of freedom and *P* value noted<br>*Give P values as exact values whenever suitable.*
☒	☐	For Bayesian analysis, information on the choice of priors and Markov chain Monte Carlo settings
☒	☐	For hierarchical and complex designs, identification of the appropriate level for tests and full reporting of outcomes
☒	☐	Estimates of effect sizes (e.g. Cohen's *d*, Pearson's *r*), indicating how they were calculated

Our web collection on statistics for biologists contains articles on many of the points above.

Software and code

Policy information about availability of computer code

Data collection	Nikon Eclipse Ti confocal system; Nikon NIS Elements (v. 5.21.00); Leica M165 FluoCombi stereoscope system; Thermo Fisher QuantStudio 5 Real Time PCR system; BioTek Epoch Plate Reader; Perkin Elmer Victor Nivo Multimode Plate Reader; MAFFT (v7, https://mafft.cbrc.jp/alignment/software/); PhyML (v3.1, http://www.phylogeny.fr/); Tree of Life (iTOL) software (v6, http://itol.embl.de), UCSC Genome Browser (genome.ucsc.edu)
Data analysis	ImageJ/Fiji software PMID:22743772 (v 1.53t); GraphPad Prism 9.5.0

For manuscripts utilizing custom algorithms or software that are central to the research but not yet described in published literature, software must be made available to editors and reviewers. We strongly encourage code deposition in a community repository (e.g. GitHub). See the Nature Portfolio guidelines for submitting code & software for further information.

Data

Policy information about availability of data

All manuscripts must include a data availability statement. This statement should provide the following information, where applicable:

- Accession codes, unique identifiers, or web links for publicly available datasets
- A description of any restrictions on data availability
- For clinical datasets or third party data, please ensure that the statement adheres to our policy

All relevant data supporting the findings of this study are available within the article and its Supplementary Information/ Source Data files. Published gene

expression datasets used in this study can be find from NCBI's Gene Expression Omnibus GSE36582 and GSE42726, Proc. Natl. Acad. Sci. USA, 10.1073/pnas.261573998, Cell Host Microbe, 10.1016/j.chom.2009.01.003, and Mbio, 10.1128/mbio.01117-14 44-48. Public databases used in this study: FlyBase (https://flybase.org), FLYATLAS 2 (flyatlas2.org), JASPER TF database (https://jaspar.genereg.net), Ensembl (https://ensemblgenomes.org/), NCBI (https://www.ncbi.nlm.nih.gov/). Source data are provided with this paper.

Research involving human participants, their data, or biological material

Policy information about studies with human participants or human data. See also policy information about sex, gender (identity/presentation), and sexual orientation and race, ethnicity and racism.

Reporting on sex and gender	N/A
Reporting on race, ethnicity, or other socially relevant groupings	N/A
Population characteristics	N/A
Recruitment	N/A
Ethics oversight	N/A

Note that full information on the approval of the study protocol must also be provided in the manuscript.

Field-specific reporting

Please select the one below that is the best fit for your research. If you are not sure, read the appropriate sections before making your selection.

☒ Life sciences ☐ Behavioural & social sciences ☐ Ecological, evolutionary & environmental sciences

For a reference copy of the document with all sections, see nature.com/documents/nr-reporting-summary-flat.pdf

Life sciences study design

All studies must disclose on these points even when the disclosure is negative.

Sample size	Pre-determining sample sizes is not necessary, as the number of Drosophila is not limited. Sample sizes were determined based on experimental variation between different genotypes and treatments. At least three independent biological replicates were carried out for each experiment.
Data exclusions	No data were excluded from these analysis.
Replication	Each experiment has been performed at least three times independently. See methods section and figure legends for details.
Randomization	All animals were randomly alloted.
Blinding	Blinding was performed in all quantifications without knowledge of genotype and/or treatment.

Reporting for specific materials, systems and methods

We require information from authors about some types of materials, experimental systems and methods used in many studies. Here, indicate whether each material, system or method listed is relevant to your study. If you are not sure if a list item applies to your research, read the appropriate section before selecting a response.

Materials & experimental systems

n/a	Involved in the study	
☐	☒	Antibodies
☒	☐	Eukaryotic cell lines
☒	☐	Palaeontology and archaeology
☐	☒	Animals and other organisms
☒	☐	Clinical data
☒	☐	Dual use research of concern
☒	☐	Plants

Methods

n/a	Involved in the study	
☒	☐	ChIP-seq
☒	☐	Flow cytometry
☒	☐	MRI-based neuroimaging

Antibodies

Antibodies used	rabbit anti-Acetylated lysine (Cell Signaling, #9441, 1:500), rabbit anti-Acetyl-Histone H3-K9/K14/K18/K23/K27 (ABclonal, #A17917, 1:500), rabbit anti-ATP5A (Abcam, #14748, 1:500), rabbit anti-phospho-Histone 3 (Cell Signaling, #9701, 1:500), mouse anti-FASN1 (Dev. Studies Hybridoma Bank #4A1, 1:10), and mouse anti-Delta (Dev. Studies Hybridoma Bank #C594.9, 1:10). Fluorescent secondary antibodies (Jackson Immunoresearch, 1:500)
Validation	1. Rabbit anti-Acetylated lysine (Cell Signaling, #9441) (https://www.cellsignal.com/products/primary-antibodies/acetylated-lysine-antibody/9441)<br>2. Rabbit anti-Acetyl-Histone H3-K9/K14/K18/K23/K27 (ABclonal, #A17917) (https://abclonal.com/catalog-antibodies/Acetyl-HistoneH3-K9K14K18K23K27RabbitpAb/A17917)<br>3. Rabbit anti-ATP5A (Abcam, #14748) (https://www.abcam.com/products/primary-antibodies/atp5a-antibody-15h4c4-mitochondrial-marker-ab14748)<br>4. Rabbit anti-phospho-Histone 3 (Cell Signaling, #9701) (https://www.cellsignal.com/products/primary-antibodies/phospho-histone-h3-ser10-antibody/9701)<br>5. Mouse anti-FASN1 (Dev. Studies Hybridoma Bank, 4A1) (https://dshb.biology.uiowa.edu/AFFN-FASN-4A1)<br>6. Mouse anti-Delta (Dev. Studies Hybridoma Bank, C594.9) (https://dshb.biology.uiowa.edu/C594-9B)<br>7. Cy™3 AffiniPure Donkey Anti-Mouse IgG (H+L) (Jackson Immunoresearch, 715-165-150) (https://www.jacksonimmuno.com/catalog/products/715-165-150)<br>8. Alexa Fluor® 488 AffiniPure Donkey Anti-Rabbit (Jackson Immunoresearch, 711-545-152) (https://www.jacksonimmuno.com/catalog/products/711-545-152)

Animals and other research organisms

Policy information about studies involving animals; ARRIVE guidelines recommended for reporting animal research, and Sex and Gender in Research

Laboratory animals	The following strains were obtained from the Bloomington Drosophila Stock Center: w1118 (#3605), Daughterless (Da)-Gal4 (#55851), UAS-nuclear localization sequence (nls)-GFP (#4775), UAS-GFP (derived from #39760), and Tubulin (tub)-Gal80 (temperature sensitive)-ts (#65406), UAS-mito-HA-GFP (#8443), UAS-Acbp3RNAi (#58343). The following strains were obtained from Vienna Drosophila RNAi Center: UAS-Acbp6RNAi (#104642), UAS-Acbp4RNAi (#109198), UAS-Acbp5RNAi (#23586), UAS-AclyRNAi (#30282); UAS-CPT1RNAi (#4046); UAS-SchlankRNAi (#33897), UAS-STAT92eRNAi (#43866); UAS-FoxoRNAi (#106097); UAS-RelishRNAi (#49413); UAS-HNF4RNAi (#12692); UAS-Nrf2RNAi (#37673); UAS-PGC1αRNAi (#103355), UAS-EgfrRNAi (#107130), UAS-dMycRNAi (#2947), and UAS-WhiteRNAi (#30033). esgGal4 was kindly provided by H. Jasper. NP1Gal4 was kindly provided by D. Ferrandon, and 10xSTAT-GFP was kindly provided by E. Bach. Upd3Gal4 and UAS-Upd3RNAi was kindly provided by N.Perrimon.<br>8-10d female adult Drosophila melanogaster were used in this study.
Wild animals	No wild animals were used in this study.
Reporting on sex	females
Field-collected samples	This study did not involve samples collected from the field.
Ethics oversight	This study did not require ethics approval.

Note that full information on the approval of the study protocol must also be provided in the manuscript.
